# Persistent Financial Burdens and High Donor Satisfaction Among Living Liver Donors: A 24-y Single-center Study

**DOI:** 10.1097/TXD.0000000000001971

**Published:** 2026-06-23

**Authors:** Piper Stacey, Ahmed Akoad, Sarah Meade, Mohamed Akoad

**Affiliations:** 1 UMass Chan Medical School, Worcester, MA.; 2 Middlebury College, Middlebury, VT.; 3 Department of Transplantation and Hepatobiliary Diseases, The Lahey Hospital and Medical Center, Burlington, MA.

## Abstract

**Background.:**

Prior investigations on living donor hepatectomy have primarily focused on medical and psychosocial outcomes, with limited evaluation of financial implications. Financial support systems exist, but these are limited by strict income criteria, leaving some donors ineligible. This study sought to characterize donor-reported quality of life with a focus on postdonation financial burden.

**Methods.:**

Over a 24-y period, 402 living donor hepatectomies were performed at a single institution. In 2024, donors were contacted by telephone and surveyed regarding their postdonation experiences, with up to 3 attempts made >3 wk.

**Results.:**

Of the 129 respondents (32%), 24 reported adverse financial consequences of donation, most commonly lost wages (n = 18). Donors reported a median absence from work of 10 wk (mean, 11.2; SD, 8.3). Additional burdens included costs of travel, lodging, and follow-up care. Six donors reported difficulty obtaining life insurance, and 2 experienced increased premiums attributable to donation-related complications. Thirty-four donors experienced medical complications attributed to donation, including hernias, gastrointestinal upset, and liver-related complications. Despite these issues, 98.4% would donate again, 97% expressed satisfaction, and 93.7% would recommend donation.

**Conclusions.:**

Living liver donors consistently reported satisfaction despite persistent financial challenges. Prospective living liver donors should be counseled during donor evaluation for the potential of financial difficulties during the donation process. Future efforts should focus on derisking the donation process by addressing nonmedical complications, such as wage loss and insurance discrimination, to better support the living donor population.

## INTRODUCTION

Previous studies have primarily focused on the medical and psychosocial outcomes of living-donor hepatectomy, with limited exploration of the financial implications for donors.

A review of 43 living liver donor quality of life publications concluded that 24% of donors faced higher-than-expected costs, 40% found donation-related expenses burdensome, and 15% still had financial concerns and life insurance changes 6 y postdonation.^1^ In an analysis of the A2ALL (Adult-to-Adult Living Donor Liver Transplantation Cohort Study), 37% of donors incurred out-of-pocket donation-related medical expenses not covered by insurance, including medical bills and medication costs.^2^ The odds of experiencing postdonation financial burdens increased when donors reported predonation concerns about who would pay donation costs, concern about missing work, lower household income, and having a technical, clerical, or lower-level position compared with a semiprofessional or professional position.^2^ Mean out-of-pocket costs are estimated to be between $6100^3^ and $9700 US dollars,^4^ accounting for inflation since the publication of these studies.^5^

It’s clear that living liver donors experience financial burdens,^1,2^ but the details of how these donors slip through the cracks of existing support systems require exploration. In 2024, 605 living liver donations occurred, potentially amounting to $5 868 500 in out-of-pocket costs incurred by living liver donors.^4,6^

While programs such as the National Living Donor Assistance Center (NLDAC) offer income reimbursement for lost wages, not all donors qualify. To be eligible, both the donor and recipient must be US citizens, and the recipient’s income must be <350% of the federal poverty guidelines—$54 775 for a single-person household in 2025. Although these criteria help to prioritize those most in need, some donors still face significant burdens. This study aimed to evaluate the quality of life of living liver donors, with a focus on postdonation financial burdens, and to identify gaps in the current support systems.

Furthermore, this study spans 24 y of living liver donation at a single center, providing a unique opportunity to evaluate financial burdens both before and after the introduction of lost wage reimbursement by NLDAC.^7^ In addition, Lahey Hospital established a Philanthropic Liver Fund in 2010 to offset travel and lodging expenses, allowing this study to assess the impact of such supplemental support on donor-reported financial outcomes.

## MATERIALS AND METHODS

All donor hepatectomy procedures performed at Lahey Hospital between 1999 and 2023 were identified. Contact was attempted via phone up to 3 times over the course of 3 wk in the summer of 2024, receiving responses from donors years after their donation. Contact information was identified using electronic medical records; however, there were cases in which this contact information changed. Donors were surveyed about their postdonation experience as well as their current health. This study was conducted under Institutional Review Board 20233089. The Institutional Review Board waived the need for consent.

For donors who reported lost wages as a financial burden after the implementation of the NLDAC wage support reimbursement program, medical charts were reviewed to determine whether they had applied for NLDAC assistance.

Data analysis was performed using Python (Version 3.9.12), NumPy (2.0.2), SciPy (1.13.1), and Matplotlib (3.9.4). Categorical variables were analyzed using chi-square or Fisher exact tests, as appropriate, and the Mann-Whitney *U* test was used to compare age distributions between survey respondents and the overall donor population at our center.

## RESULTS

**FIGURE 1.**
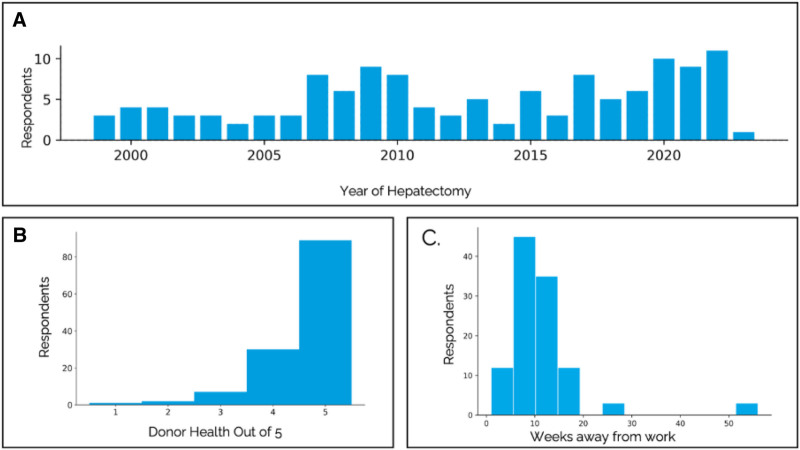
Summary of donor survey responses across our center’s 24-y living donor liver transplantation program. A, Number of donor responses by year of donor hepatectomy. Donor responses spanned our center’s 24-y history of living donor hepatectomies. B, Donor-reported health score out of 5 at the time of survey completion. C, Weeks taken off work after hepatectomy (median = 10, SD = 8.3).

A total of 129 donors responded to the survey, yielding a response rate of 32% across the entire study period (Figure 1A). Respondents differed from nonrespondents in several demographic characteristics. Most notably, the proportion of male donors was significantly lower among respondents (45% versus 55%; *P* = 0.010; Table 1). No differences were observed in graft laterality (83% versus 82%; *P* = 0.99) or donor race (*P* = 0.96). Hispanic ethnicity was more common among respondents than nonrespondents (9.4% versus 4.6%), reaching borderline statistical significance (*P* = 0.050). Twelve respondents (9.3%) were altruistic living donors who previously did not know the recipient. When asked to rate their current health out of 5, donors reported an average health score of 4.6, indicating that most donors considered themselves to be quite healthy (Figure 1B).

**TABLE 1.** Respondent and nonrespondent demographics

Donor demographics	Donors who responded (n = 129)	Donors who did not respond (256)	*P*
Donor male sex, n (%)	55 (45)	146 (55)	0.01
Right lobe donated, n (%)	88 (83)	173 (82)	0.99
Donor age at transplant, mean ± SD	41 ± 10.7	37 ± 10.27	0.067
Donor race, n (%)			0.96
White	107 (88)	217 (82)	
Black	1 (0.8)	3 (1)	
Asian	3 (2.4)	8 (3)	
Other	8 (6)	16 (6)	
Ethnicity			
Hispanic, n (%)	11 (9.4)	9 (4.6)	0.05

### Complications After Donation

Thirty-four donors reported complications attributed to their donation (Table 2). Complications were categorized into major (n = 2) and minor (n = 32) severity (Table 2). Digestive disturbances were the most frequently cited issue (12 donors), followed by abdominal discomfort (6 donors). Incisional hernias occurred in 7 donors.

**TABLE 2.** Complications attributed to living liver donation by donors

Complication	Patients (n)	Description	Financial burden, n (%)	Complication severity
Abdominal discomfort	6	Numbness, incisional pain	1 (16)	Minor
Digestive issues	12	Bloating, diarrhea, upset stomach, weight gain	1 (8.3)	Minor
Hernia	7	Incisional hernias	2 (28.6)	Minor
Metabolic/endocrine	2	Diabetes	0 (0)	Minor
Liver-related	4	1 Cirrhosis, 1 fatty liver, 1 ascites, 1 elevated LFTs	4 (100)	2 Minor (ascites and transient elevation in LFTs), 2 major (fatty liver of the graft remnant, cirrhosis)
Musculoskeletal/other	2	1 Fibromyalgia, 1 herniated disc	0 (0)	Minor
None	96	–	16 (16.7)	–

LFT, liver function tests; –, not applicable

Four donors reported liver-related complications (Table 2). One donor developed portal hypertension and ascites without evidence of cirrhosis despite extensive evaluation. Another donor developed cirrhosis of the remnant liver 10 y following donation with no clear pathogenesis but without clinical consequences. One donor was diagnosed with fatty liver disease with preserved liver function, and another donor developed transient elevation of transaminases 11 mo postdonation that later normalized. The patients who developed cirrhosis of the remnant liver and fatty liver disease were classified as having major complications.

Liver-related complications were strongly associated with financial strain, with all affected donors reporting related costs. In contrast, among the 96 donors who reported no complications, 16.8% still experienced some form of financial burden.

### Financial Burdens

**FIGURE 2.**
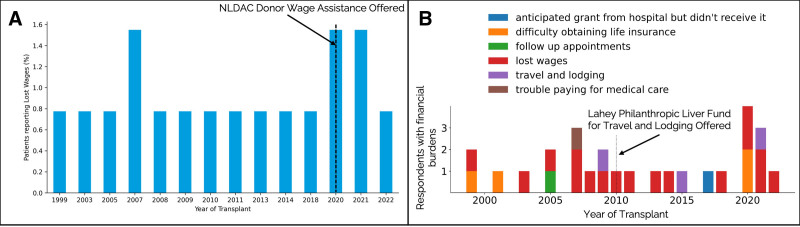
Reported financial burdens among living liver donors. A, Percentage of respondents reporting financial burdens per year. NLDAC donor wage assistance was implemented in November 2020 (black dotted line). Years without reports of wage-related financial burdens were excluded. B, Types of financial burdens reported over time. The Lahey Philanthropic Liver Fund for travel and lodging expenses was introduced in 2010 (gray dotted line). NLDAC, National Living Donor Assistance Center.

Among the 24 living liver donors reporting financial challenges from donation, 32 burdens were reported (Table 3). Lost wages were the most common burden, accounting for over half of the reports (56.3%, n = 18), followed by difficulty obtaining life insurance (18.8%, n = 6). Other reported burdens included travel and lodging costs (9.4%), increased insurance premiums (6.3%), trouble paying for medical care (3.1%), unfulfilled hospital grants (3.1%), and costs related to follow-up visits (3.1%). Donors reported a median time away from work of 10 wk, with a SD of 8.3 wk (Figure 1C). Among donor professions with >2 respondents, nurses (n = 8) and teachers (n = 7) were the most frequently represented (Table 4). The mean time away from work by profession varied substantially, ranging from an average of 4.9 wk among self-employed donors to 52 wk for a single respondent in banking. Nurses had the longest mean leave among groups with multiple responses (19.5 ± 13 wk), while teachers and managers reported shorter average leave times (8.7 ± 3.6 and 10.3 ± 1.7 wk, respectively). Financial burdens were most commonly reported by donors in sales (50%, 2/4) and technicians (33%, 2/6), while only 12.5% of nurses and 14.3% of teachers and managers reported financial strain. Donors reported financial burdens throughout the history of our living donor hepatectomy program (Figure 2A). Even after the implementation of NLDAC wage assistance in 2020, 0.75%–1.5% of donors continued to experience lost wage-related burdens. Wage-related burdens did not decrease after the implementation of wage support through NLDAC. Similarly, despite our center’s 2010 introduction of the Philanthropic Liver Fund to offset travel and lodging expenses, some donors still reported these costs as burdensome (Figure 2B). One donor specifically noted anticipating a travel grant that had never been received.

**TABLE 3.** Financial burdens attributed to donation (some donors reported >1 type of burden; n = 32 total reports, 24 donors)

Type of financial burden	No. of reports, n (%)	White race, n (%)
Lost wages	18 (56.3)	13 (72)
Difficulty obtaining life insurance	6 (18.8)	6 (100)
Travel and lodging costs	3 (9.4)	3 (100)
Increased insurance premiums	2 (6.3)	1 (50)
Trouble paying for medical care	1 (3.1)	1 (100)
Anticipated grant from hospital but did not receive it	1 (3.1)	1 (100)
Costs related to follow-up visits	1 (3.1)	1 (100)

**TABLE 4.** Donor professions with >2 respondents

Profession of donor	No. of donors	Mean weeks away from work, n (±SD)	Donors experiencing financial burdens, n (%)
Nurse	8	19.5 (±13)	1 (12.5)
Teacher	7	8.7 (±3.6)	1 (14.3)
Manager	7	10.3 (±1.7)	1 (14.3)
Technician	6	12.0 (±2.1)	2 (33)
Police officer	5	14.4 (±7.5)	1 (20)
Self-employed	4	4.9 (±2.0)	1 (25)
Administrator	4	8.8 (±2.4)	–
Sales	4	11.0 (±3.6)	2 (50)
Banking	3	52 (±0)^*a*^	–
Insurance	3	12.0 (±0)	–
Military	3	9.2 (±4.0)	–
Firefighter	3	10.7 (±1.9)	–

^*a*^Only 1 respondent who worked in banking responded to the question regarding mean time away from work.

Overall, donor satisfaction with living liver donation was high (Table 5). Nearly all respondents indicated that they would donate again (98.4%) and would still have chosen to donate knowing their recovery experience (98.4%). A majority (93.7%) would recommend living liver donation to others, and 82.9% remained registered organ donors after their donation.

**TABLE 5.** Donor satisfaction

Question	% Yes
Would you recommend living liver donation to someone else?	93.7
Would you donate again?	98.4
Are you a registered organ donor after your donation?	82.9
If you knew about the recovery, would you still have donated?	98.4

## DISCUSSION

This study evaluated the postdonation experiences of 129 living liver donors, providing valuable insights into the persistent financial challenges that they face, even in the presence of established support systems. While our findings reaffirm the high levels of satisfaction and altruism consistently reported in the literature, they also highlight the critical gaps in financial support that disproportionately affect certain donors. The study found that 34 of the 129 respondents experienced medical complications attributed to living liver donation. The most frequently reported issues were digestive disturbances and abdominal discomfort (Table 2). While complications such as eating and weight changes, metabolic issues, and musculoskeletal problems were also reported, liver-related complications—including current liver disease, fatty liver, and transient elevated liver function tests—were most strongly associated with financial burden, as 100% of these 4 donors reported associated costs. The prevalence of medical complications at our center is in line with other studies; however, the percentage of patients reporting an associated financial burden has not yet been reported in the literature.^8,9^ Despite the presence of these medical issues, the vast majority of donors still expressed high levels of satisfaction and would donate again, consistent with previous studies.^9,10^ The vast majority of donors rate their current health as “quite healthy” (or 4/5). The high willingness (98.4%) to donate again despite knowing the complications likely reflects the self-selected, highly motivated donor population who undergo extensive psychological screening and are driven to help a loved one.

Lost wages were the most common financial burden reported by 18 donors, which aligns with previous research that highlights the substantial economic impact of time away from work.^1,2^ The median work absence of 10 wk highlights the prolonged recovery associated with living liver donation and underscores the importance of lost wage support. Certain professions, including nurses and technicians, reported both longer leave durations and higher rates of financial strain, suggesting occupational disparities in access to paid leave or employer-based support, consistent with previous studies.^2^ Currently, our center advises donors to plan for approximately 8 wk away from work ; however, data from this study (median = 10 wk, mean = 11.2, SD = 8.3) may help set more realistic expectations and improve financial preparedness for potential donors.

The findings related to financial support programs are particularly noteworthy. The data show that financial burdens, including lost wages and travel costs, continued to be reported at the same rate even after the implementation of the NLDAC wage reimbursement in 2020 and the Lahey Philanthropic Liver Fund in 2010 (Figure 2).^7^ This suggests that while these programs are well-intentioned, they may not be fully addressing the needs of all donors. The restrictive income criteria of NLDAC, for example, may leave patients who are donating to middle- and high-income recipients ineligible for assistance, even if these donors experience significant financial hardship from lost wages or other out-of-pocket costs.

We examined the 3 donors who reported lost wage-related financial burdens after the implementation of the NLDAC wage support program. Two donors were eligible for assistance: 1 did not complete the application, potentially attributable to language barriers, and required 12 wk off from construction work; the other received reimbursement but still experienced financial strain, requiring 7 wk off from work. The third donor was ineligible because the recipient’s income exceeded 350% of the federal poverty line; this donor, the recipient’s son, worked part-time in carpentry and required 16 wk off. This case highlights the limitations of determining donor wage support based solely on recipient income.

Furthermore, travel and lodging costs remained persistently burdensome despite the implementation of our center’s Philanthropic Liver Fund. One donor’s specific report of not receiving an anticipated grant further illustrates the potential for administrative or logistical failure in these systems. However, there is no record in the electronic health record of this donor asking about or receiving information about this grant.

Future research should aim to assess the demographics of donors who experience financial burdens but do not qualify for NLDAC. Qualitative research, such as in-depth interviews, could provide a richer understanding of the specific barriers and experiences that donors face. Additionally, future studies should more precisely assess financial losses, particularly across varying levels of complications. Ultimately, our findings underscore the need for institution-specific assessments of donor support mechanisms to ensure that the financial burdens of this life-saving gift do not create a barrier to equitable access to living donation.

### Addressing the Broader Disparities

The study also sheds light on other financial challenges beyond lost wages. Six donors reported difficulty obtaining life insurance, and 2 experienced increased premiums, which can have long-lasting financial consequences. These findings suggest that the impact of donation extends beyond the immediate recovery period, affecting donors’ long-term financial stability and ability to plan for the future. The demographic distribution of the financial burden provides important insights. Although most donors reporting financial challenges were White, this reflects the overall racial composition of our donor population. Notably, only 72% of donors reporting lost wages were White, compared with 88% of the total respondents, suggesting that donors from other racial or ethnic groups may be disproportionately affected by lost wages. Additionally, 1 Spanish-speaking donor did not apply for NLDAC wage reimbursement despite receiving instructions in Spanish, highlighting potential barriers to access. These findings underscore the need for equitable and accessible support systems that address the diverse needs of all potential donors.

Potential policy solutions include expanding donor financial assistance beyond recipient income, standardizing wage replacement protections, and safeguarding against increased life insurance premiums after donation. We also implemented these findings at our center by discussing the potential for lost wages and financial difficulties in addition to the medical and surgical complications during the consent process.

### Limitations and Future Directions

Our study has several limitations. The 32% response rate may reflect response bias, as donors with particularly positive or negative experiences may have been more likely to participate. Follow-up was limited by outdated contact information, nonresponse, and, in some cases, inability to update donor records after recipient death. Nonetheless, respondents span all years of living donor liver transplantation at our center and represent diverse professions. Efforts are underway to maintain more up-to-date donor contact information going forward.

The reliance on self-reported data is also a limitation, as financial burdens may be underreported because of stigma or may be difficult to quantify accurately. Additionally, our study was conducted at a single institution, which limits the generalizability of our findings. Furthermore, as data were collected years to decades after donation, recall bias may affect the accuracy of reported complications and financial burdens, and donors may perceive these outcomes differently when reflecting retrospectively than they would have in the immediate postdonation period. However, patient-reported complications in our study were present throughout our center’s history of transplantation (**Figure S1**, **SDC**, https://links.lww.com/TXD/A876).

## Supplementary Material


