# Supplementary figures and images for: Persistent Financial Burdens and High Donor Satisfaction Among Living Liver Donors: A 24-y Single-center Study
Source: Transplant Direct. 2026 Jun 23;12(7):e1971. doi: 10.1097/TXD.0000000000001971 (PMC13340708; doi:10.1097/TXD.0000000000001971)

### Complication Categories by Year of Transplant

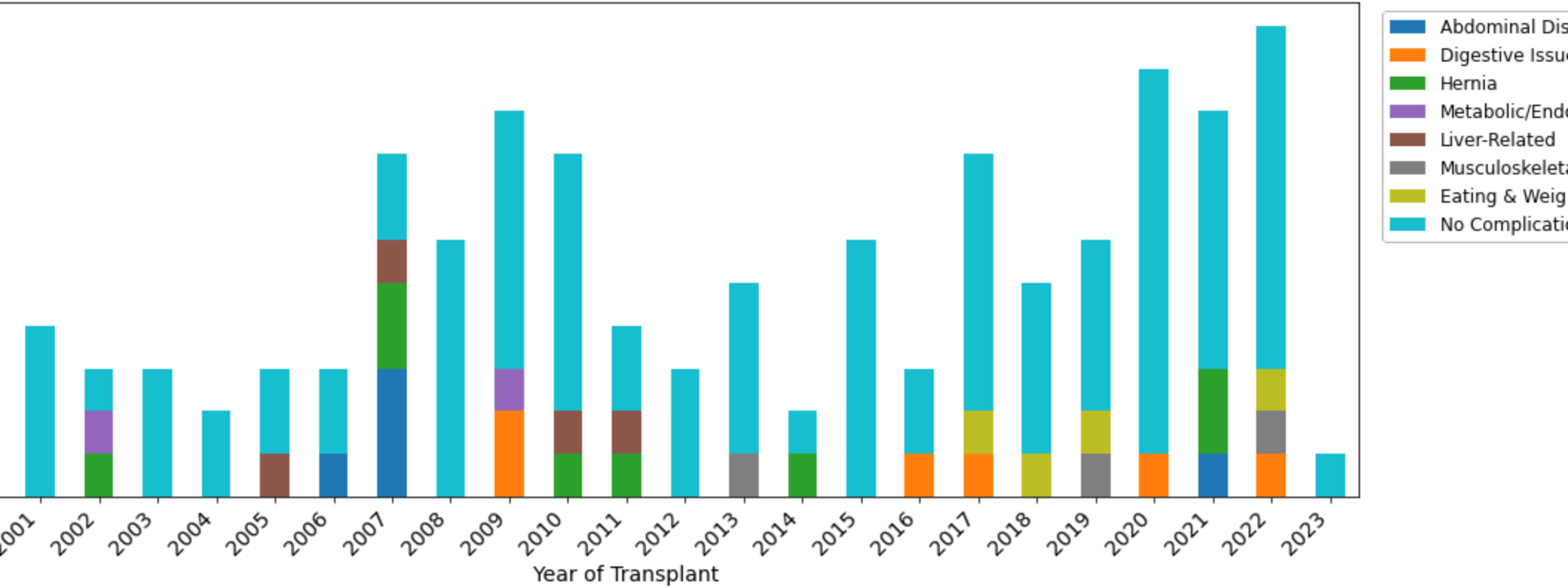

Supplement: Supplementary file 1 [file txd-12-e1971-s001.pdf]
